# Differential replication properties among H9N2 avian influenza viruses of Eurasian origin

**DOI:** 10.1186/s13567-015-0198-8

**Published:** 2015-07-06

**Authors:** Rokshana Parvin, Awad A. Shehata, Kristin Heenemann, Malgorzata Gac, Antje Rueckner, Mohammad Y. Halami, Thomas W. Vahlenkamp

**Affiliations:** Institute of Virology, Center for Infectious Diseases, Faculty of Veterinary Medicine, University of Leipzig, 04109 Leipzig, Saxony Germany; Department of Pathology, Faculty of Veterinary Science, Bangladesh Agricultural University, Mymensingh, Bangladesh; Avian and Rabbit Diseases Department, Faculty of Veterinary Medicine, University of Sadat City, Sadat City, Egypt

## Abstract

Avian influenza H9N2 viruses have become panzootic in Eurasia causing respiratory manifestations, great economic losses and occasionally being transmitted to humans. To evaluate the replication properties and compare the different virus quantification methods, four Eurasian H9N2 viruses from different geographical origins were propagated in embryonated chicken egg (ECE) and Madin-Darby canine kidney epithelial cell systems. The ECE-grown and cell culture-grown viruses were monitored for replication kinetics based on tissue culture infectious dose (TCID_50_), Hemagglutination (HA) test and quantitative real time RT-PCR (qRT-PCR). The cellular morphology was analyzed using immunofluorescence (IF) and cellular ELISA was used to screen the sensitivity of the viruses to amantadine. The Eurasian wild type-H9N2 virus produced lower titers compared to the three G1-H9N2 viruses at respective time points. Detectable titers were observed earliest at 16 h post inoculation (hpi), significant morphological changes on cells were first observed at 32 hpi. Few nucleotide and amino acid substitutions were noticed in the HA, NA and NS gene sequences but none of them are related to the known conserved region that can alter pathogenesis or virulence following a single passage in cell culture. All studied H9N2 viruses were sensitive to amantadine. The G1-H9N2 viruses have higher replication capabilities compared to the European wild bird-H9N2 probably due to their specific genetic constitutions which is prerequisite for a successful vaccine candidate. Both the ECE and MDCK cell system allowed efficient replication but the ECE system is considered as the better cultivation system for H9N2 viruses in order to get maximum amounts of virus within a short time period.

## Introduction

Avian influenza H9N2 is a low pathogenic avian influenza virus (LPAIV) which in many countries continues to cause respiratory diseases, drop in egg production and increase in mortality among commercial domestic poultry and wild birds [1-3]. H9N2 viruses become endemic in poultry in many Eurasian countries particularly in some Asian and Middle Eastern countries [4]. Molecular genetic analyses of H9N2 viruses, isolated during the last two decades revealed that these viruses are highly evolving and a genetically diverse population [5]. Furthermore, H9N2 viruses have reassorted with other avian influenza subtypes to generate multiple novel subtype [6-10]. Additionally, its extensive species tropism, distribution and ability to donate internal genes to the highly pathogenic H5 and H7 subtypes [11-14] evoke particular concerns. The H9 viruses are now also being considered as potential pandemic threats, as they have acquired human virus-like receptor specificity [15] and are able to be directly transmitted from birds to humans [16]. Two distinct lineages of H9N2 influenza viruses, the North American lineage and the Eurasian lineage, have been defined. The Eurasian H9N2 viruses further grouped into three sub-lineages: G1, Y280 and Kr-p96323 based on their antigenic and genetic properties [17]. The G1-H9N2 viruses are widespread and more likely affected in commercial poultry flocks with moderate clinical signs whereas, Y280 and Kr-p96323 are apparently circulating in natural reservoir hosts and more prevalent in their respective origin areas. Multiple clades of H9N2 viruses such as G1 and Y280 have been circulating together in China [18]. Additionally, H9N2 co-circulating with other subtypes especially H5N1 in many countries raised the possibility of multiple reassorted viruses. Moreover, human infection with H9N2 viruses was observed and some of them belonged to the sub-lineage G1 [13,19,20]. Currently the biological properties and risk assessment studies of different clades of H9N2 viruses were performed in animal models [5]. Assessing the fitness of distinct clades of H9N2 showed that the North American H9N2 virus had the lowest risk profile while the Eurasian viruses displayed various levels of fitness across individual assays [5].

Due to the continuous outbreaks of H9N2 in the several mentioned countries, the development of laboratory techniques for efficient isolation and detection in surveillance samples continue to be of high priority. Upon receiving a field sample, virus propagation and isolation are important for the recovery and production of a viable viral stock for further laboratory use. The embryonated chicken egg (ECE) and cell culture systems are generally the basic choice for influenza virus cultivation. ECEs are considered the gold standard method of isolation as they are able to support the growth of a large spectrum of AIVs and their subtypes. The advantage of the ECE system is the possibility to acquire a large volume of the viral stock [21] from a single egg, thus, influenza vaccines have traditionally been prepared in ECEs [22]. Effective AIV isolation can also be performed in the cell culture system. The Madin-Darby canine kidney (MDCK) is the cell line of choice for AIV propagation and is also recommended by the World Health Organization [23]. After successful propagation, another important observation is the virus quantification. Virus quantification presents a rate-limiting step at many stages of vaccine development and production, for both egg and cell culture. Currently, one of the most widely used tools for the determination of virus concentration is the viral plaque assay, or variations such as tissue or egg culture infectious dose (TCID_50_/EID_50_). The viral plaque assay or TCID_50_/EID_50_ is a subjective and traditional biological technique that was originally applied to the quantification of viruses in the early 1950s [24]. For influenza viruses, the hemagglutination (HA) assay is also widely applied. The HA assay is rapid, requires the use of animal red blood cells, and yields an HA titer value that is not readily translated into viruses per mL. Another currently used method for virus quantification includes quantitative real time PCR (qRT-PCR). Although the qRT-PCR method does not take into account the infectious properties of the virus rather can also detect defective virus particles, it is widely used by many researcher.

In this study, four H9N2 isolates from different sources (Three G1-H9N2 and one European wild bird-H9N2) were propagated separately in two recommended effective biological systems. The virus quantification was carried out based on TCID_50_, HA as well as qRT-PCR simultaneously from all the viruses grown on both systems. The morphological changes of the embryos and cells at different time points during propagation were observed. Furthermore, the genetic evolution of the viruses in a single replication cycle in cell culture was analyzed as well as all four H9N2 viruses were checked for amantadine sensitivity. The aim of this study involved the following objectives; i) Replication efficiency of the H9N2 viruses of different origin, ii) Growth kinetics of the viruses in two different biological system, iii) Correlation between different viral quantification methods iv) Amantadine sensitivity and adaptive mutation of the viruses.

## Materials and methods

### Viruses and cells

Four Eurasian lineage H9N2 viruses: A/chicken/Bangladesh/VP01/2006 (BVP01), A/turkey/Germany/R869/2012 (GR869), A/chicken/Saudi Arabia/R61/2002 (SAR61) and A/chicken/Dubai/F5/2013 (DF5) were used in this study. Genetically, The BVP01 [14], SAR61 and DF5 [25] belong to G1 lineage, whereas GR869 is an Eurasian wild bird group (not published). The sequences accession number and details molecular analysis were available in recently published article [14,25].

Madin-Darby canine kidney cells (both the parental MDCK [ATCC® CCL-34™] and its clone MDCK-II [ATCC® CRL-2936™]) were used for efficient viral propagation of the above mentioned viruses.

### Virus propagation in ECE

Specific pathogen free chicken eggs (VALO BioMedia GmbH, Germany) were used for the ECE propagation system. Firstly, the H9N2 viruses were inoculated blindly into the allantoic cavity route of 10-days-old ECE and incubated at 37 °C. Allantoic fluids (AFs) were harvested upon the death of the embryo or at 72 h post inoculation (hpi). The presence of virus was confirmed by HA and subjected to titration. Viral EID_50_ titers were determined by injecting 100 μL of 10-fold dilutions of the virus into the allantoic cavities of 10- days-old eggs. For each dilution four eggs were used for accurate calculation of the titer. The 50% end points were calculated according to the method of Reed and Muench [26] for 50% egg infectious dose (EID_50_) and are expressed in log_10_ EID_50_/mL. The virus stock containing the titer of 7.5 log_10_ EID_50_/mL was further inoculated into embryonated chicken eggs. Three eggs at each incubation period of 2, 8, 16, 24, 32, 40, 48, 56 and 72 h were selected and AFs were harvested for the assessment of replication kinetics.

### Virus propagation in cell culture

Confluent monolayers of MDCK and MDCK-II cells were maintained in cell growth medium (CGM) consisting of Dulbecco’s modified Eagle’s medium (DMEM) supplemented with 10% fetal calf serum (FCS), 1% Na-pyruvate and 1% non-essential amino acids (NEAA). Cells were cultured in T-75 cm^2^ flasks and incubated at 37 °C in the presence of 5% CO_2_. For virus inoculation, confluent monolayer of cells was maintained in T-25 cm^2^ flask as well as in 6-well and 96-well microplates for different purposes. The inoculum was prepared by diluting the virus in growth medium (GM) [CGM without FCS and supplemented with TPCK-trypsin (2 μg/mL)] at a multiplicity of infection (moi) of 0.2 and inoculated onto the monolayer of cells. Infected cells were incubated at 37 °C for 1 h to allow viral adsorption. Afterwards the inoculum was removed and GM was added to the monolayer. The supernatants were harvested at 2, 8, 16, 24, 32, 40, 48, 56 and 64 hpi and stored at −80 °C for virus titration.

### Assessment of viral replication kinetics

Viral replication kinetics was monitored for infectious particles by tissue culture infectious dose (TCID_50_) assay, HA assay as well as for viral particles by qRT-PCR targeting the Matrix (M) gene. The influence of H9N2 viruses on cellular morphology was assessed using immunofluorescence.

#### Hemagglutination assay

The HA assay was performed using 1% washed chicken red blood cells (RBCs) prepared in PBS according to the OIE manual [27]. The harvested AFs and cell culture supernatants (CCSs) at selected time points were tested for hemagglutinating activity. The titers were recorded to draw the virus replication kinetics and were expressed by hemagglutination titer unit (HAU). Briefly, 25 μL of undiluted AF/CCS were added to the first wells of 96-well V- bottom shaped plates containing 25 μL/well of PBS. Serial two-fold dilutions were performed followed by addition of 25 μL PBS to each well. Subsequently, 25 μL of 1% washed chicken RBCs were added to each well and the plates were incubated for 45 min at room temperature (RT). The titration was read until the highest dilution giving complete agglutination and presented as log_2_ HAU/25 μL.

#### TCID_50_ assay

To determine the infectivity titer, 2 × 10^4^ cells were seeded in 96-well microplates. The harvested virus from each incubation period of both propagation systems was subjected to 10-fold serial dilutions. Six replicates of 100 μL diluted inoculum were transferred to the monolayers of MDCK-II cells and allowed to adsorb at 37 °C for 1 h. The inoculum was discarded and 150 μL of GM was added to all wells containing monolayers. The cells were incubated at 37 °C, 5% CO_2_ incubator for 32 h. The plates were observed for cytopathic effect (CPE) and the presence of virus until a certain dilution was confirmed by immunofluorescence microscopy. The viral titer was calculated as log_10_ TCID_50_/mL as described by Karber-Spearman [28,29].

#### Immunofluorescence

The 96-well microplates containing MDCK-II cells were inoculated with the virus and incubated for different time periods. Following incubation cells were washed 3 times with PBS, fixed with 3.7% formaldehyde for 10 min at RT and permeabilized with 90% ice cold methanol for 15 min. The plates were washed twice with PBS followed by addition of 3% bovine serum albumin (BSA) in PBS as a blocking solution and incubated at RT for 30 min. Plates were washed again twice and treated with influenza rabbit anti-nucleoprotein (NP) polyclonal primary antibody (PA5-32242, Thermo scientific, Germany) at a dilution of 1:5000. The treated plates were incubated at 37 °C for 1 h and washed twice with PBS. Secondary antibody (Alexa Fluor® 488 Goat Anti-Rabbit IgG, Life technologies, Germany) at 1:1000 dilutions was added and incubated again for 1 h at 37 °C. Final washing was performed twice and the plates were left to dry at RT. To investigate the morphological changes of the cell nucleus, an additional stain, 4, 6-diamidino-2-phenylindole (DAPI), was used. The plates were observed under fluorescence microscope (Olympus IX70) and images were captured for analysis.

#### Quantitative real time PCR (qRT-PCR)

RNA was isolated from both AFs and CCSs using QIAmp Viral RNA mini kit (QIAGEN, Hilden, Germany) without using carrier RNA provided by the kit and reverse transcribed under the standard conditions of RevertAid Reverse Transcriptase (Thermo Scientific, Germany) using the Uni 12 primer [30]. Diluted cDNA (1:10) was used for the PCR reaction. A unique primer pair was designed to amplify the target sequence of the M gene of AIV type A generating a product of 150 bp (primer sequences are available on request). The qRT-PCR reactions were carried out according to the manufacturer’s instructions using the Rotor-Gene SYBR Green PCR master mix (QIAGEN, Hilden, Germany). A pJET 1.2 BVP/ H9N2-M plasmid was used as a positive control to develop a standard curve. The genome copy number was calculated based on the standard curve and expressed as log_10_ genome copies/reaction.

### Adaptive mutations (HA, NA and NS genes)

To determine the viral adaptive mutations in cell culture, the hemagglutinin (HA), neuraminidase (NA), non-structural (NS) and polymerase basic (PB1 & PB2) genes were sequenced and analyzed after a single passage. Full length standard RT-PCR was performed and the PCR products were purified using GeneJET Gel Extraction Kit (Thermo Scientific, City, Germany). The purified products were subjected to direct nucleotide sequencing using Rhodamin Dye-Terminator Cycle Sequencing Ready Reaction Kit (Big Dye ® Terminator v1.1; Applied Biosystem), followed by analysis in an ABIPRISM™ 310 genetic Analyser (Applied Biosystems). The sequence data were edited aligned and analyzed using three software packages (DNASTER, BioEdit and MEGA).

### Screening of amantadine sensitivity

The sensitivity of the studied H9N2 viruses to amantadine was assayed in MDCK-II cells according to previously described method with modifications [31]. Briefly, 10^4^ TCID_50_ of the studied H9N2 influenza viruses were tested against concentrations of 0, 2.5 and 7.5 μg/mL amantadine (Sigma Aldrich, Steinheim, Germany) in 96 well microtiter plates by checkerboard titration. The MDCK-II monolayer was washed and pre-incubated with each drug concentration in GM for 30 min. In a parallel plate, each titer of the virus isolate was incubated with the two different drug concentrations in GM for 1 h at RT. The drug-containing medium in the pre-incubated cell culture plate was replaced by drug treated virus solution from a parallel plate and further incubated for 1 h at 37 °C. Plates were washed and GM containing the desired concentration of the drug was added. Plates were incubated at 37 °C for 24 h. Following the removal of medium, plates were washed with PBS and fixed with 90% methanol overnight at −20 °C. Subsequently, endogenous peroxidase activity was blocked by incubation with 3% (v/v) H_2_O_2_ for 1 h followed by blocking in PBS containing 0.05% Tween-20 and 3% bovine serum albumin. Primary NP antibody was added and incubated at RT for 1 h. After washing with PBS containing 0.05% Tween-20, IgG HRP anti-rabbit antibody (Thermo scientific, Germany) at 1:1000 dilutions was added and the plates were incubated 1 h at RT. Freshly prepared substrate (Dako, Denmark) was added and incubated for 20 min at RT in the dark. Stop solution (H_2_SO_4_, 2.5 M) was added and the absorbance OD of the wells was read at 450/650 nm. The mean OD of the infected cultures without drug was equal to the total amount of NP protein (100%). Cultures infected with amantadine-sensitive virus should produce less than 50% of the total NP protein.

## Results

### Comparison of hemagglutination (HA) titer

**Figure 1:**
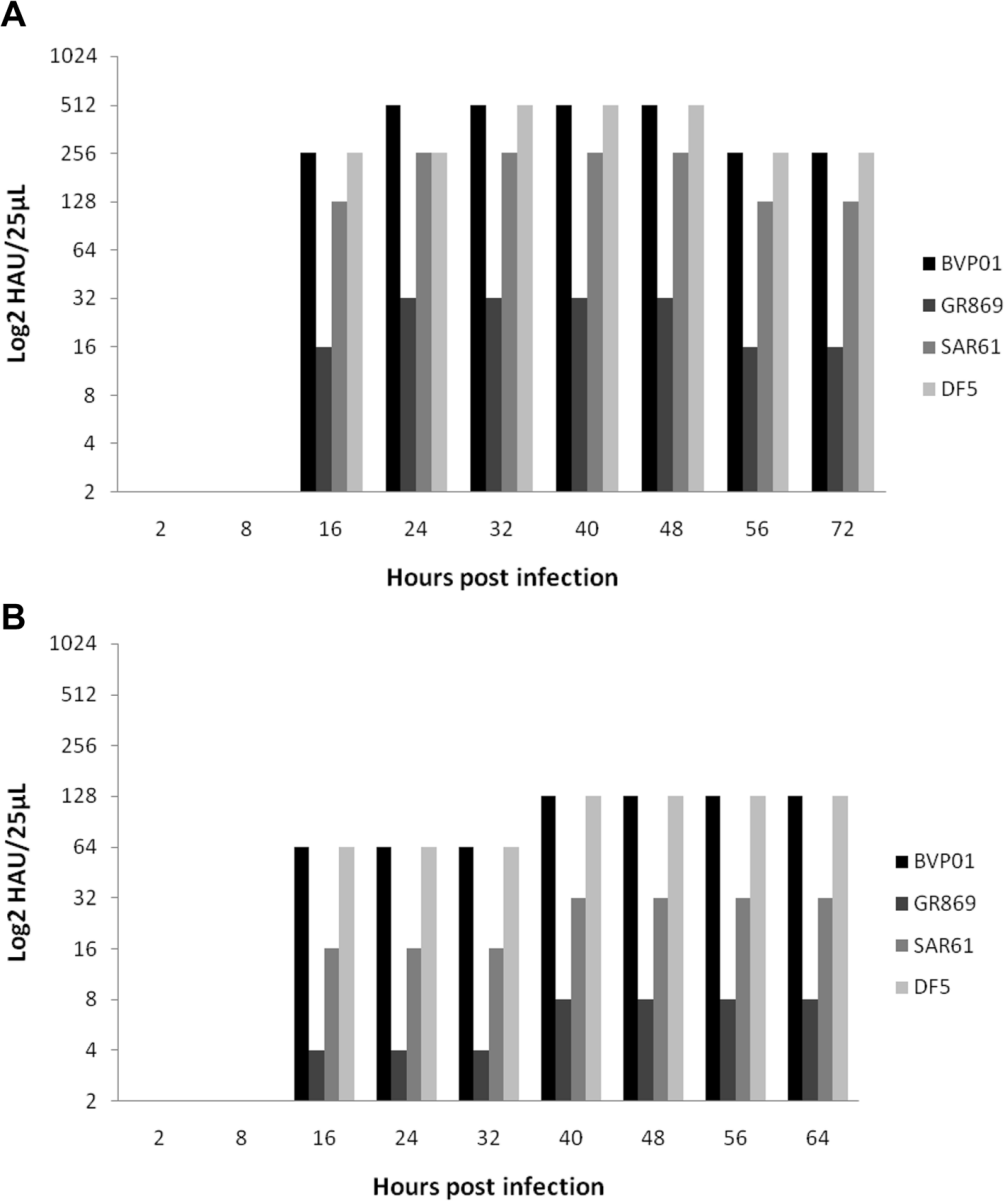
**H9N2 growth curve based on hemagglutination unit titer.** Comparison of the hemagglutination unit titer of the four studied H9N2 viruses grown in embryonated chicken eggs (**A**) and MDCK-II cells (**B**) at different time points.

The HA titer was calculated both from the harvested AFs and CCSs of ECE- grown and cell culture-grown viruses, respectively. All tested H9N2 viruses did not show any differences in titers in MDCK and MDCK-II cells, thus only a diagram representative for MDCK-II is shown (Figure 1B). Most viruses started yielding HAU earliest at 16 hpi and reached maximum yield within 32 to 48 hpi which continued until 64 to 72 hpi (Figure 1). In the ECE system the currently circulating G1 lineage viruses BVP01, DF5 and SAR61 showed higher yields than the European wild type GR869. The highest titers reached by BVP01, DF5, SAR61 and GR869 9log_2_ (512), 9log_2_ (512), 8 log_2_ (256) and 5 log_2_ (32), respectively (Figure 1A).

On the other hand, in cell culture propagation system the highest titers recorded by BVP01, DF5, SAR61 and GR869 were 7log_2_ (128), 7log_2_ (128)_,_ 5log_2_ (32) and 3log_2_ (8)_,_ respectively (Figure 1B). The ECE-grown viruses produced two-fold higher virus yield than the cell culture-grown viruses.

### Replication kinetics based on TCID_50_

**Figure 2:**
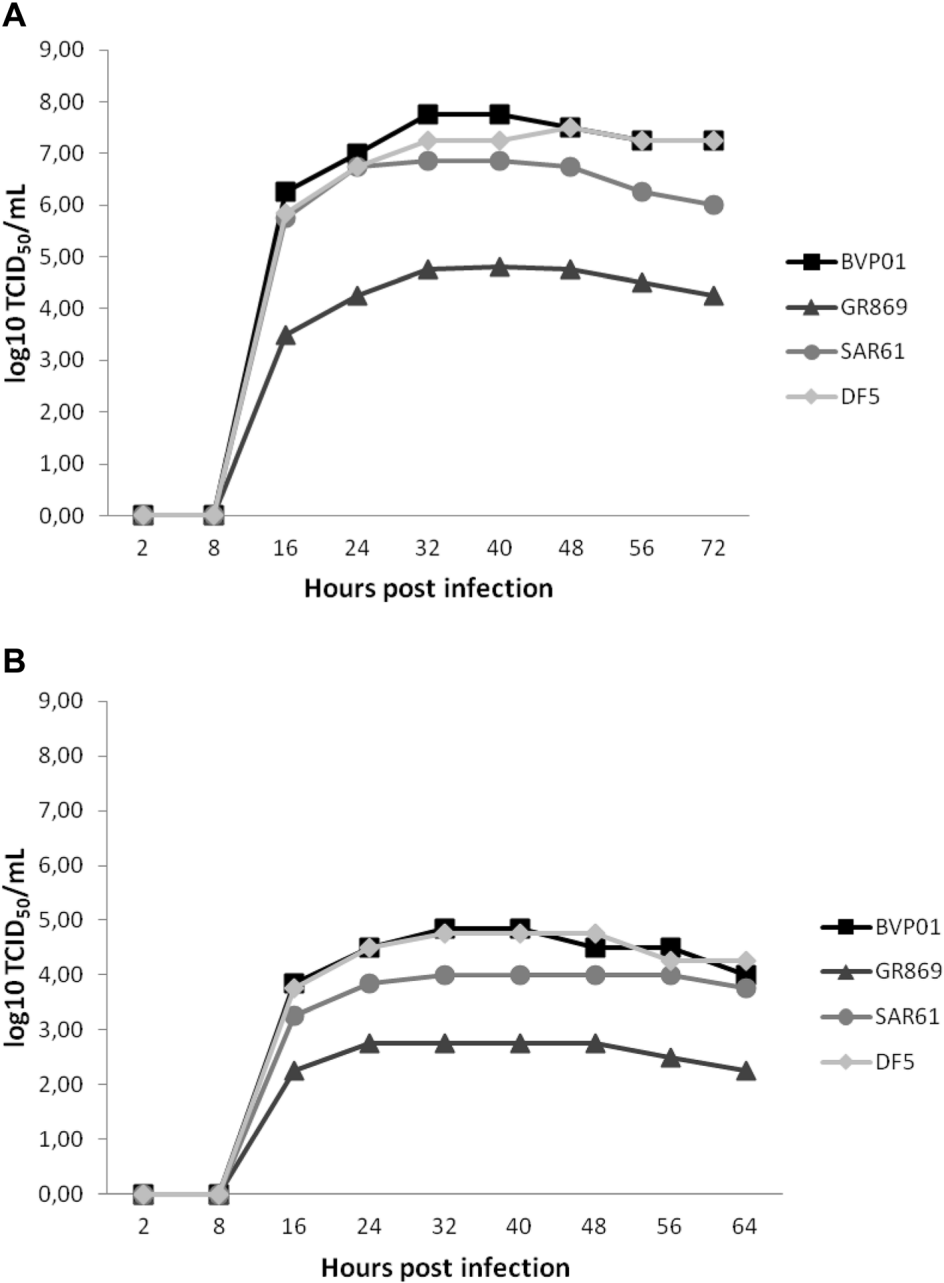
**H9N2 growth curve based on TCID**_**50**_ **titer.** Replication kinetics of the four studied H9N2 viruses grown in embryonated chicken egg (**A**) and MDCK-II cells (**B**) at different selected time points.

TCID_50_ count was performed in MDCK-II cells to quantify the virus infectivity and to compare kinetics among the H9N2 viruses grown in the both systems. The ECE- and cell culture-grown H9N2 viruses showed detectable titers at 16 hpi and reached maximum titers at 32 hpi (Figure 2). The infectivity titers of all ECE-grown H9N2 viruses varied between 3.5 log_10_ TCID_50_/mL to 7.8 log_10_ TCID_50_/mL. The ECE-grown BVP01 and DF5 viruses reached maximum yields (~8 log_10_ TCID_50_/mL) followed by SAR61 (~7 log_10_ TCID_50_/mL) and GR869 (~5 log_10_ TCID_50_/mL) (Figure 2A).

The infectivity titers of cell culture-grown H9N2 strains varied between 2.3 log_10_ TCID_50_/mL to 4.9 log_10_ TCID_50_/mL. Furthermore, the cell culture-grown BVP01, SAR61 and DF5 viruses reached maximum yields (>4 log_10_ TCID_50_/mL) in contrast to GR869 (2.8 log_10_ TCID_50_/mL) (Figure 2B). Thus, the BVP01, SAR61 and DF5 viruses grown in both the biological systems achieved maximum titers compared to the GR869. Furthermore, all ECE-grown viruses showed higher virus yields than the cell culture-grown viruses.

### Comparison of viral genome copy number

**Figure 3:**
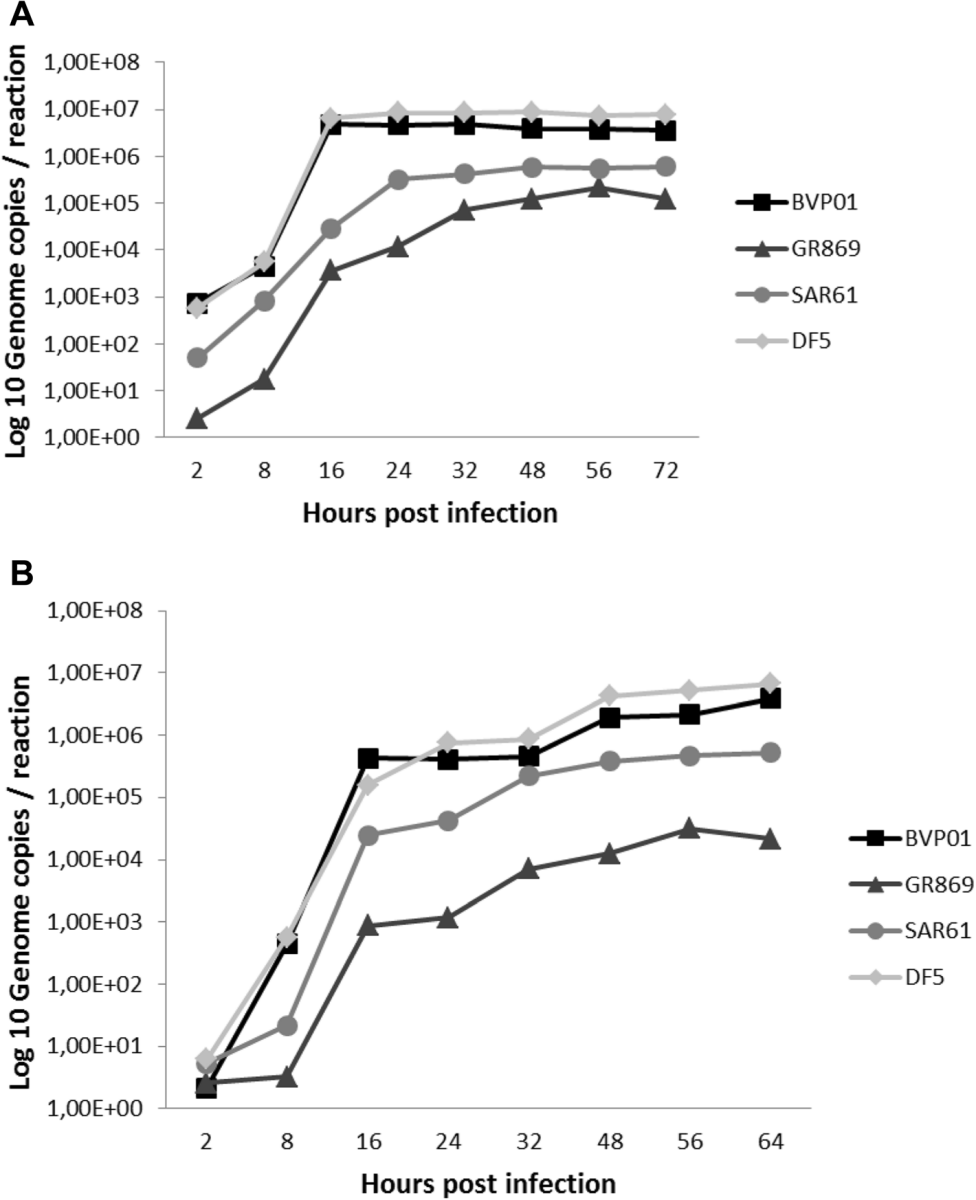
**H9N2 growth curve based on M genome copies.** Comparison of the four studied H9N2 virus grown in embryonated chicken egg (**A**) and MDCK-II cells (**B**) based on the measurement of M genome copies at different time points.

ECE and MDCK-II systems were capable of supporting efficient viral replication and did not measured significant differences in genome copy number based on the M gene (Figure 3). The ECE-grown BVP01 (3 log_10_ genome copies/reaction), DF5 (3 log_10_ genome copies/reaction) and SAR61 (~2 log_10_ genome copies/reaction) viruses showed detectable copy number earliest at 2 hpi. The maximum copy number (~7 log_10_ genome copies/reaction) was obtained earliest at 16 hpi for BVP01 and DF5 H9N2 (Figure 3A) whereas, the maximum copy number (~5.5 log_10_ genome copies/ reaction) for SAR61 was detected earliest at 48 hpi. The European wild type GR869 ECE-grown H9N2 showed detectable copy numbers earliest at 8 hpi (1 log_10_ genome copies/reaction) and reached the maximum (~5 log_10_ genome copies/reaction) earliest at 48 hpi (Figure 3A).

On the other hand, cell culture-grown BVP01, DF5, SAR61 and GR869 viruses exhibited detectable copy numbers earliest at 8 hpi. The maximum copy number of BVP01 and DF5 was >6 log_10_ genome copies/reaction, detected at 48 hpi. Whereas, the SAR61 showed maximum ~5 log_10_ genome copies/reaction also at 48 hpi and the GR869 showed maximum ~4.3 log_10_ genome copies/reaction at 56 hpi (Figure 3B).

### Morphology and progression of infection

**Figure 4:**
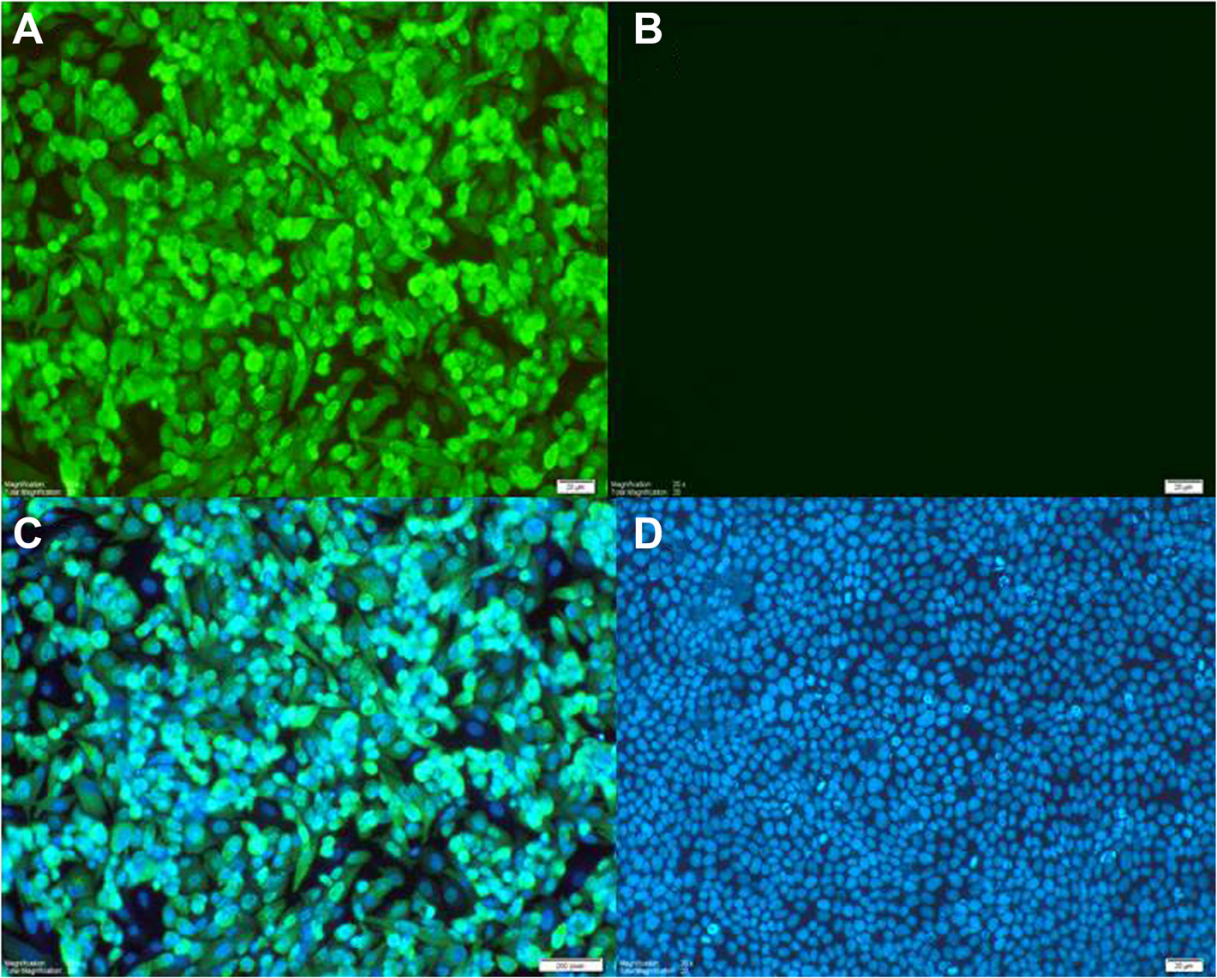
**Detection of influenza virus H9N2 in infected cells.** Confirmation of the presence of influenza BVP01/H9N2 virus at 32 hpi in MDCK-II cells observed by immunofluorescence assay (magnification 20×) using influenza A anti-NP antibodies. **A**: Infected cells positive for NP protein; **B**: Mock control negative for NP staining; **C**: Merge between NP and DAPI staining on infected cells and **D**: Mock control positive for DAPI staining.

**Figure 5:**
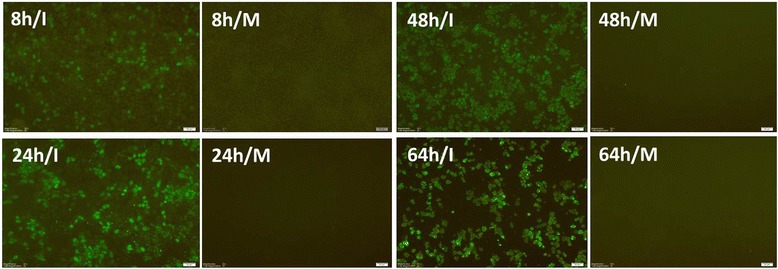
**Morphological changes of infected cells.** Progression of infection and viral replication in MDCK-II cell at different time points of BVP01/H9N2 virus infection as observed by immunofluorescence assay (magnification 20×) using influenza A anti-NP antibodies. I: Infected cells positive for NP protein and M: Mock control negative for NP staining.

The general morphology and spread of ECE- and cell culture-grown H9N2 viruses were monitored at the specified time points. In ECE propagation, most embryos were alive until the last time point chosen in this experiment. However, some embryos showed nonspecific mortality earliest at 32 hpi when infected with the same virus and dose, which might be due to the adverse viral influence on the embryos. In the cell culture system, both MDCK and MDCK-II cell lines showed similar morphology during infection; therefore, only the MDCK-II-derived growth morphology is shown. Firstly, one selected virus (BVP01) was confirmed at 32 hpi in MDCK-II cells to check the antibody, selected stains and to adjust the suitable control (Figure 4). On the growth kinetics, the viruses showed CPE in inverted microscope observation earliest at 16 hpi, although the viral replication was detected at 8hpi when tested against nucleoprotein (NP) of AIV in IF stain (Figure 5). CPE was more extensive at 48 to 64 hpi and >90% cells have detached by 72 hpi. Additionally, on TCID_50_ count, the presence of virus by producing CPE was confirmed at the 2^nd^ dilution while the virus was actually present until the 5^th^ dilution as confirmed by IF stain. Thus, the TCID_50_ titers were considered not only based on CPE but also considering the highest dilution where NP protein was recognized by IF stain. The progression of viral infection in MDCK-II cells varied in morphology at different time points (Figure 5). Influenza NP protein was detected by IF at the studied time points suggestive of a high permissibility and efficient spread of virus infection in the cells. At the early time points of infection the viral NP protein was mostly detected in the nucleus and later disseminated to the cytoplasm. Significant damage and loss of nuclear structure of the cells at later time points was clearly observed by DAPI stain alone. At 64 hpi, very few cells remained attached to the culture plates.

### Sequence analysis and mutation

The HA, NA, NS, PB1 and PB2 genes play important roles in host-virus interactions and virulence. Thus, the respective gene sequences of cell culture-grown viruses were compared to the original sequences of the stock virus (AF stock passage) used as inoculum. The analysis of the HA, NA and NS genes revealed 2-6 nucleotide mutations resulting in 1-2 amino acid substitutions depending on the respective genes (Table 1), whereas the polymerase genes remain similar as inoculum. Importantly, none of these nucleotide or amino acid mutations was observed in the conserved region, which could have altered pathogenesis or virulence. No amino acid changes were observed at the HA cleavage site, Receptor binding site and within the N-glycosylation sites of the HA protein. All of the viruses possessed their respective motif in the PDZ domain at the C terminal end of the NS1 protein as inoculum.

**Table 1:** Sequence analysis after a single passage of the viruses in MDCK-II compared with the sequence from the AF stock inoculum

Viruses	HA		NA		NS	
	nt	aa	nt	aa	nt	aa
BVP01	6	G270R	1	no	1	NS1:S27L
		T279I				NS2: no
GR869	3	no	no	no	1	NS1: Q142E
						NS2: no
SAR61	2	no	no	no	1	NS1: no
						NS2: R77K
DF5	5	no	1	no	no	NS1: no
						NS2:no

nt: Nucleotide, aa: Amino acid, no: No substitutions.

### Amantadine sensitivity

The drug sensitivity of all four studied H9N2 viruses was confirmed using in situ ELISA in MDCK-II cells. All tested viruses were shown to be sensitive to amantadine. Amantadine (2.5 μg/mL or 7.5 μg/mL) inhibited the growth of all viruses at titers of 4 log_10_ TCID_50_/mL. The presence of NP at amantadine concentration of 2.5 μg/mL was 45%, 34%, 26%, and 55% for BVP01, DF5, GR869 and SAR61, respectively. At amantadine concentrations of 7.5 μg/mL the presence of NP was accordingly 28%, 34%, 20% and 45%.

## Discussion

Efficient isolation and propagation of influenza viruses is important in epidemiological surveillance, study of host-pathogen interactions, diagnosis and vaccine production. Successful and efficient propagation of H9N2 virus on ECE and cell culture system depends on viral dose or moi, molecular genetic properties of the virus, receptor binding properties of the host cell and some other virus- or host-related factors [32-34]. The main focus of this study was to evaluate efficient H9N2 virus propagation in two different biological systems. Thus, four different geographical sources of H9N2 viruses were propagated in ECEs as well as MDCK and MDCK-II cell lines. Analyses of the replication kinetics, correlation between the virus quantification methods, virus adaptations to cell culture and the sensitivity to amantadine of the H9N2 viruses were performed.

The replication of influenza virus in a host cell is a polygenic process depending on the host cell endocytic pathways for entry and transfer of viral genome as well as activation of host cell signaling [35,36]. Preferentially, avian influenza viruses bind to terminal α-2,3 SA linkage, whereas human influenza bind to α−2,6 SA. The allantoic cells of ECEs contain α−2,3 SA Gal while the amniotic cells of ECEs and MDCK cells contain both linkages [37]. Therefore, the allantoic cavities are considered to be the preferential sites for avian influenza virus. In this study, four H9N2 viruses were propagated and replication kinetics were measured based on HA titer, TCID_50_ titer and qRT-PCR for genome (M) copies. The replication ability of the four virus strains were assessed separately in both systems. The four H9N2 viruses were found to grow efficiently in ECEs and cell lines. In the cell culture system, both parenteral MDCK and cloned MDCK-II cells were used however; both cell lines have shown the same morphology and pattern of replication kinetics. Thus, only MDCK-II yields were shown in this study. The cell culture-grown viruses exhibited 2-3 fold lower virus titers than the ECE grown virus titers, however, in genome copy number it was 1-1.5 fold lower than the ECE grown viruses. Although the qRT-PCR method does not take into account the infectious properties of the virus rather can also detect defective virus particles. Among the four studied viruses, the G1 H9N2 (BVP01, DF5 and SAR61) isolates achieved maximum virus yields in contrast to the European wild bird (GR869) isolate based on HA titer, TCID_50_ titer as well as on genome copy count.

Generally, all the analyzed strains grew well in both propagation systems, however, the maximum virus yields and the earliest time points to achieve highest titers varied with the propagation systems as well as with individual viruses. The ECE system allowed rapid replication and yielded maximum titers within 16-32 h, whereas cell culture supported relatively slow replication and yielded maximum titers after 48 h. In addition, the choice of different moi on virus replication is an important issue [34]. The moi of 0.2 used in this study for cell culture, was found to be relatively high as inoculum to generate maximum yields at longer time points. More than 80% cells were found detached in both the cell lines after 64 hpi. Thus, the ECE-propagated viruses reached relatively higher virus titers compared to cell culture-propagated viruses at respective time points. Therefore, the ECE may be considered the better system for the primary virus isolation from field samples as well as for the virological surveillance study. Specific receptor binding properties of embryos also facilitate better replication of avian origin influenza virus compared to cell lines [38]. The ECE and cell culture are completely different biological systems which possess differential host-related factors involved in efficient viral propagation as well as in variation of viral replication kinetics.

The G1 lineage H9N2 viruses are adapted to poultry and produced more severe infections than the other representative lineage of H9N2. Thus, the genetic background of all studied H9N2 viruses were also considered for achievement of different replication kinetics at both propagation systems. Genetically, the BVP01, DF5 and SAR61 (not published) belonged to the G1 lineage, while GR869 is an Eurasian wild-type reassortant virus (not published) isolated from turkey which was not adapted to domestic poultry flocks. Moreover, the genetic variation at HA cleavage motif, HA receptor binding site (RBS) and C terminal domain of NS1 protein (Table 2) of four studied viruses thought to be involve in achievement of different replication pattern or in virus particle count from two propagation systems. The binding property of the virus to the host cell is determined by two factors, the RBS affinity of the virus and receptor density on the host cell surface [39]. The RBS motif of the HA protein of the BVP01 and DF5 revealed an important Q234L (Glutamine to Leucine) mutation, like the HK-G1strain. However, the SAR61 and GR869 viruses contain Q at 234 positions (H3 numbering: 226) which is a typical avian virus signature, and it has been reported that the presence of this aa results in a preference for binding to α 2,3-linked sialic acid (avian receptors) whereas viruses having L234 (H9 numbering) showed a preference for 2,6-linked sialic acid (human receptors) besides avian receptor and a potential cause of reported human infections [40].

**Table 2:** Molecular genetic background of genome regions important for viral replication

Virus	HA cleavage site	Right pocket 146−150	Left pocket 232−237	NA stalk deletion	NS1-C domain	M2 blocker				
						26	27	30	31	34
A/chicken/Bangladesh/ VP01/2006	PAKSSR*GLF	GTSKS	NGLIGR	No	KSEV	L	V	A	S	G
A/turkey/Germany/R869/2012	PAASGR*GLF	GTSKA	NGQQGR	No	ESEV	L	V	A	S	G
A/chicken/Saudi Arabia/R61/2002	PARSSR*GLF	GTSKS	NGQQGR	No	EPEV	L	V	A	S	G
A/chicken/Dubai/ F5/2013	HARSSR*GLF	GTSKS	NGLIGR	No	GSEV	L	V	A	N	G

Due to be the member of a separate clade with different HA cleavage motif, RBS as well as NS1C-terminal domain (Table 2) the GR869 virus showed comparatively lower replication pattern than the other three strains of G1 H9N2. Again it was noticed that within the three studied G1 H9N2 viruses, the BVP01 and the DF5 produced kinetics were somewhat similar with higher replication profile in contrast of SAR61 which showed lower kinetics. Epidemiologically, BVP01 and DF5 viruses were known to induce clinical signs and caused mortality in commercial poultry flocks which gives the back draw to yield highest titers in all the virus quantification methods taken into account in this study from both the propagation systems.

Amantadine blocks the ion channel formed by M2 protein and inhibits the early step of replication. Substitutions of amino acid residue such as L26F, V27A, A30T, S31N and G34E of the M2 protein are already known to confer resistance to the amantadine [41,42]. The amino acid residue analysis of M2 protein of all studied H9N2 viruses except one position in DF5 H9N2 (had substitution S31N), did not showed any substitutions on those mentioned residues (Table 2). However, at the amantadine screening on MDCK-II cells at two different concentrations confirmed all the studied H9N2 were sensitive to the amantadine M2 blocker drug. The nucleotide sequences of HA, NA and NS gene from cell culture-grown viruses did exhibit some mutations compared to the sequence of the inoculum. However, none of these substitutions were found to alter molecular determinants of pathogenesis.

In conclusion, the virus replication kinetics based on HA titer, TCID_50_ titer and the viral genome copies revealed that the three G1-H9N2 viruses (BVP01, DF5 and SAR61) reached highest kinetic level compared to the European wild type H9N2 (GR869) virus probably due to the variable genetic constitutions at their specific conserved region. The species variation of the GR869 virus may have also affected the replication profile. The virus quantification methods used in this study have a correlation among themselves as the ECE-grown and cell culture-grown viruses achieved similar pattern of replication kinetics from all the quantification methods. However, this study revealed that the ECE propagation system allowed better replication as maximum virus yield were more than the cell culture-grown viruses. Thus, the replication efficiency of the ECE-grown H9N2 viruses was greater probably due to the specific binding property between the virus and the host cell. The different virus quantification methods varied insignificantly. All the studied viruses were amantadine sensitive and did not exhibit any significant mutations that are required for the alteration of pathogenesis after a single replication cycle. This study adds more insights in the growth properties of the Eurasian lineage of avian influenza H9N2 in two traditional effective propagation systems which further help in figuring out a suitable system for the influenza vaccine production.
